# Human Choline Kinase-α Promotes Hepatitis C Virus RNA Replication through Modulation of Membranous Viral Replication Complex Formation

**DOI:** 10.1128/JVI.00960-16

**Published:** 2016-09-29

**Authors:** Mun-Teng Wong, Steve S. Chen

**Affiliations:** Institute of Biomedical Sciences, Academia Sinica, Taipei, Taiwan, Republic of China; University of Southern California

## Abstract

Hepatitis C virus (HCV) infection reorganizes cellular membranes to create an active viral replication site named the membranous web (MW). The role that human choline kinase-α (hCKα) plays in HCV replication remains elusive. Here, we first showed that hCKα activity, not the CDP-choline pathway, promoted viral RNA replication. Confocal microscopy and subcellular fractionation of HCV-infected cells revealed that a small fraction of hCKα colocalized with the viral replication complex (RC) on the endoplasmic reticulum (ER) and that HCV infection increased hCKα localization to the ER. In the pTM-NS3-NS5B model, NS3-NS5B expression increased the localization of the wild-type, not the inactive D288A mutant, hCKα on the ER, and hCKα activity was required for effective trafficking of hCKα and NS5A to the ER. Coimmunoprecipitation showed that hCKα was recruited onto the viral RC presumably through its binding to NS5A domain 1 (D1). hCKα silencing or treatment with CK37, an hCKα activity inhibitor, abolished HCV-induced MW formation. In addition, hCKα depletion hindered NS5A localization on the ER, interfered with NS5A and NS5B colocalization, and mitigated NS5A-NS5B interactions but had no apparent effect on NS5A-NS4B and NS4B-NS5B interactions. Nevertheless, hCKα activity was not essential for the binding of NS5A to hCKα or NS5B. These findings demonstrate that hCKα forms a complex with NS5A and that hCKα activity enhances the targeting of the complex to the ER, where hCKα protein, not activity, mediates NS5A binding to NS5B, thereby promoting functional membranous viral RC assembly and viral RNA replication.

**IMPORTANCE** HCV infection reorganizes the cellular membrane to create an active viral replication site named the membranous web (MW). Here, we report that human choline kinase-α (hCKα) acts as an essential host factor for HCV RNA replication. A fraction of hCKα colocalizes with the viral replication complex (RC) on the endoplasmic reticulum (ER) in HCV-infected cells. NS3-NS5B expression increases ER localization of wild-type, but not D288A mutant, hCKα, and hCKα activity facilitates the transport of itself and NS5A to the ER. Silencing or inactivation of hCKα abrogates MW formation. Moreover, hCKα is recruited by NS5A independent of hCKα activity, presumably through binding to NS5A D1. hCKα activity then mediates the ER targeting of the hCKα-NS5A complex. On the ER membrane, hCKα protein, *per se*, induces NS5A binding to NS5B, thereby promoting membranous RC formation and viral RNA replication. Our study may benefit the development of hCKα-targeted anti-HCV therapeutics.

## INTRODUCTION

More than 170 million people worldwide are infected with hepatitis C virus (HCV), and persistent HCV infection may result in progression to life-threatening diseases, including cirrhosis and hepatocellular carcinoma. HCV is an enveloped virus classified in the genus *Hepacivirus* within the family Flaviviridae (1, 2). This virus has a 9.6-kb single-stranded RNA genome with positive polarity flanked by 5′ and 3′ untranslated regions (UTRs) (2). Translation of the HCV genomic RNA produces a polyprotein that undergoes further processing by cellular and viral proteases into structural proteins (core, E1, and E2) and nonstructural (NS) proteins (p7, NS2, NS3, NS4A, NS4B, NS5A, and NS5B) (1, 2). The structural proteins assemble into the viral particle, whereas the NS proteins play crucial roles in genome RNA replication and virion assembly (1, 2).

Similar to many other positive-sense RNA viruses, HCV hijacks host lipids and remodels the endomembrane system to create a lipid-rich environment necessary for viral replication (3). The viral replication complex (RC), also called the replicase, is composed of viral proteins NS3 to NS5B and the replicating viral RNA (4). These viral RCs are housed on altered endoplasmic membranes and form distinct organelle-like structures termed membranous webs (MWs) (5–8). These MWs are characterized by their unique multivesiculated membrane vesicles, which have heterogeneous sizes, ranging between 100 to 300 nm in diameter, and morphologies and which are embedded within a subcellular membrane structure (9, 10). Immunogold electron microscopy (EM) showed that all viral proteins formed a complex that associated with the NS4B-induced MW (5). The MW serves as a platform for compartmentalizing and concentrating the HCV RC, viral products, and host factors to ensure efficient viral replication and assembly (2, 11).

Among the NS proteins, NS3 is a bifunctional protein that has serine-type protease, NTPase, and helicase activities, whereas NS4A acts as a cofactor for NS3 protease. NS4B, an integral membrane protein, is thought to serve as the scaffold for viral RC assembly and is able to induce MW formation (12, 13). Within the RC, the viral RNA-dependent RNA polymerase NS5B transcribes viral genome RNA (2). NS5A is a multitasking viral protein that is present as two phosphorylated forms: hypophosphorylated p56 and hyperphosphorylated p58 (14). Possessing an RNA-binding ability (15), NS5A contains an N-terminal amphipathic helix (AH) that tethers the protein to the membrane (16), three domains, i.e., D1, D2, and D3, and two low-complexity sequences, LCS1 and LCS2, which are located in between the domains (12, 17, 18). D1 functions in RNA replication and is associated with lipid droplet (LD) and NS5A dimerization (19, 20). LCS1 and D2 function in RNA replication (12), while D3 plays a critical role in the NS5A-core protein interaction and virion assembly (21, 22).

LD serves as not only a host lipid storage site but also a dynamic organelle in HCV replication and pathogenesis (23, 24). NS5A is thought to facilitate the transport of viral RNA from the MW replication site to LDs to interact with core protein, thereby promoting viral RNA encapsidation, nucleocapsid formation, and virus assembly (24–26). In addition, LDs are tightly associated with the E1- and E2-localizing ER membrane within the MW, where viral RNA replication occurs (24, 27). Thus, the close proximity of LDs, the MW, and the endoplasmic reticulum (ER) membrane effectively couples viral RNA replication to virion assembly.

**FIG 1:**
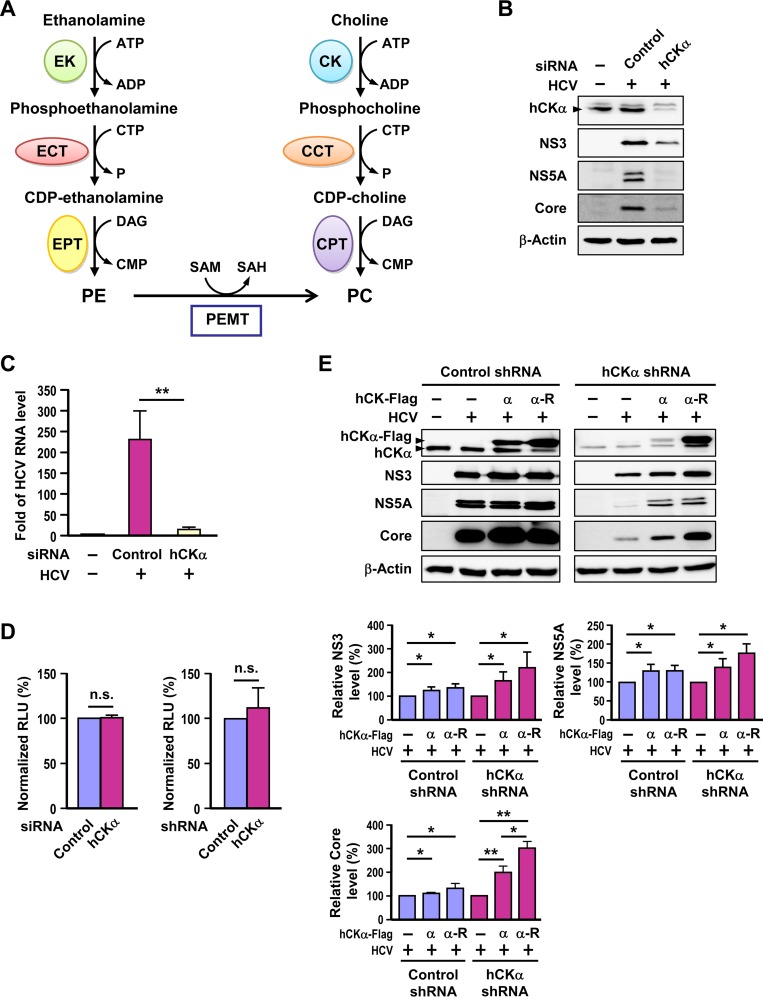
Involvement of hCKα in HCV replication. (A) Biosynthesis of phosphatidylcholine (PC) and phosphatidylethanolamine (PE). PC is mainly synthesized via the CDP-choline and PE methyltransferase (PEMT) pathways. In the CDP-choline pathway, choline kinase (CK), CTP:phosphocholine cytidylyltransferase (CCT), and choline phosphotransferase (CPT) catalyze consecutive reactions to produce PC. In the PEMT pathway, PE undergoes three methylation reactions catalyzed by PEMT to synthesize PC. In the CDP-ethanolamine pathway, ethanolamine is taken up into the cytoplasm and catalyzed by ethanolamine kinase (EK), CTP:phosphoethanolamine cytidylyltransferase (ECT), and ethanolamine phosphotransferase (EPT) to yield PE. (B and C) Huh7 cells remained untransfected (−) or were transfected with an untargeted control siRNA or hCKα-specific siRNA. Cells were then infected with HCVcc at an MOI of 1 or received parallel treatment without HCVcc (−). Cells were then subjected to Western blot analysis (B) and RT-PCR (C). (D) Huh7 cells transfected with control or hCKα siRNAs (left panel) and Huh7 cells stably transduced with control or hCKα shRNA lentiviral vectors (right panel) were assessed for cell viability. (E) The control and hCKα shRNA Huh7 stable cells were transfected with plasmids encoding the Flag-tagged normal hCKα (α) or shRNA-resistant hCKα (α-R) followed by infection with HCV. The cells were analyzed by Western blotting (top panel). The levels of NS3, NS5A, and core proteins in HCV-infected control or hCKα stable knockdown cells overexpressing hCKα or hCKα-R are expressed as a percentage relative to that detected in vector plasmid transfected cells, which was designated 100% (bottom panel). *, *P* < 0.05; **, *P* < 0.01; ns, nonsignificant.

Human choline kinase-α (hCKα), the first enzyme in the CDP-choline (or Kennedy) pathway (28), catalyzes the phosphorylation of choline into phosphocholine (Fig. 1A). Phosphocholine is converted to CDP-choline by CTP:phosphocholine cytidyltransferase (CCT) (Fig. 1A); this conversion is the rate-limiting step in the CDP-choline pathway. CDP-choline is then processed by CDP-choline:1,2-diacylglycerol cholinephosphotransferase (CPT) to phosphatidylcholine (PC) (Fig. 1A), the most abundant phospholipid in eukaryotic cell membranes (29). As essential components of the cell membrane, PC and phosphatidylethanolamine (PE) serve as precursors of the lipid second messengers involved in cell survival and growth (30). CKα induces the G_1_-to-S phase transition of the cell cycle and affects many genes involved in cell proliferation, transformation, and signaling transduction (31). CKα overexpression is oncogenic (32). Tissues derived from most common human tumors show higher levels of CKα expression and enzymatic activity than their normal counterparts, and high levels of CKα are associated with high histological tumor grades and poor clinical outcomes (33, 34). Silencing of hCKα by small interfering RNA (siRNA) or treatment with *N*-(3,5-dimethylphenyl)-2-{[5-(4-ethylphenyl)-1H-1,2,4-triazol-3-yl]sulfanyl} acetamide (CK37), a specific inhibitor of hCKα activity, leads to decreased phosphocholine and PC levels in transformed and neoplastic human cells and prevents tumor formation in mice (35, 36) and in a human breast cancer xenograft (37).

In an siRNA screen of the human kinome to identify host factor requirements for HCV replication, Reiss et al. noticed that silencing of CKα exhibited the strongest inhibitory effect on HCV replication among the 13 kinases screened (38). Li et al. also performed screening experiments and found that CKα is a critical factor for HCV entry and viral RNA replication (39). Nevertheless, whether hCKα itself or the CDP-choline pathway is important for HCV replication in hepatoma cells and how hCKα engages with HCV to regulate viral replication remain elusive.

In the present study, we show that hCKα itself, through its enzymatic activity, not the CDP-choline pathway, positively modulates HCV RNA replication. We also show that hCKα is recruited by NS5A, likely through the binding of hCKα to D1 of NS5A. In addition, hCKα acts concertedly with NS5A to cooperatively facilitate targeting of the hCKα-NS5A complex to the ER in an hCKα activity-dependent manner. On the ER membrane, the hCKα protein, not hCKα activity, functions as a mediator for NS5A-NS5B binding, thereby promoting functional viral RC assembly and MW formation crucial for viral RNA replication.

## MATERIALS AND METHODS

### Cell cultures and antibodies.

Human hepatoma cell lines Huh7 and Huh7.5-1 and human embryonic kidney 293T cells (ATCC CRL-3216) were cultured as previously reported (40). The following mouse monoclonal antibodies (MAbs) were purchased: anti-core protein (sc-57800; Santa Cruz); anti-NS3 (MAB8691 [Merck Millipore] and ab65407 [Abcam]); anti-NS5A (HCM-131-5; Austral Biologicals); anti-NS5B (HCV-4B8; BioFront Technologies); anti-Flag M2, anti-Myc, and anti-β-actin (F1804, M4439, and A5441, respectively; Sigma-Aldrich); anti-green fluorescent protein (GFP) and anti-hemagglutinin (HA) (Gm0001-01 and M0003, respectively; Abomics); and anti-human cluster differentiation 81 (CD81) and anti-calnexin (555675 and 610523, respectively; BD Biosciences). The following rabbit polyclonal antibodies were purchased: anti-hCKα (ab88053; Abcam); anti-tail-interacting protein of 47 kDa (TIP47) (sc-14726; Santa Cruz); anti-phosphatidylethanolamine *N*-methyltransferase (PEMT), anti-Flag, and anti-HA (SAB1401522, F7425, and H6908, respectively; Sigma-Aldrich); anti-glyceraldehyde 3-phosphate dehydrogenase (GAPDH), anti-CCTα, anti-Myc, and anti-green fluorescent protein (GFP) (GTX100118, GTX62359, GTX115046, and GTX113617, respectively; GeneTex); anti-binding immunoglobulin protein (BiP) (3177S; Cell Signaling Technology); and normal IgG (12-370; Merck Millipore). The purchased goat antibodies included anti-calregulin (CALR) and anti-adipose differentiation-related protein (ADRP) (sc-6467 and sc-32450, respectively; Santa Cruz). The MAb 9E10 was directed against NS5A (41). Alexa Fluor-conjugated secondary antibodies were obtained from Invitrogen.

### Reagents and siRNAs.

OptiPrep (1114542) was purchased from Axis-Shield. CK37 (CAS1001478-90-5) was purchased from Merck Millipore. Hexadecyltrimethylammonium bromide (HDTAB; H5882), hexadecylphosphocholine (HePC; M9198), and 5-aminoimidazole-4-carboxamide 1-β-d-ribofuranoside (AICAR; A9978) were obtained from Sigma-Aldrich. Small interfering RNAs (siRNAs) targeting hCKα (HSS140690 and HSS140691), CCTα (HSS107689, HSS107690, and HSS107691), and CD81 (HSS101629, HSS101630, and HSS101631) were purchased from Invitrogen, while a nontargeting control siRNA (29551) was obtained from Santa Cruz.

### Plasmids and construction.

The plasmids pUC-JFH1 and pFL-Luc-JFH1 were as previously described (40, 42). pSGR-Luc-JFH1 (where SGR is subgenomic replicon) (43), pWPI-T7-BLR, and pTM-NS3-5B (44) were as previously described. A set of pCDNA3.1(−)/Myc-His-based plasmids expressing full-length NS5A or D1, D2, or D3 of NS5A was as previously described (45). Plasmids expressing HA-tagged genotype 1b Con1 strain NS proteins and the plasmid expressing HA-tagged glutathione *S*-transferase (GST) were as previously described (46). Plasmids pCMVΔR8.91 and pMD.G and the pLKO-based short hairpin RNA (shRNA) constructs TRC0005 and TRCN0000284352 were obtained from the National RNAi Core Facility at Academia Sinica. pCMV6-hCKα (RC207209), which encodes hCKα tagged with Myc and DDDK (or Flag) tags at the C terminus, was purchased from Origene, while pEGFP-C1-hCKα encodes hCKα fused with EGFP at the N terminus.

The pCMV6-hCKα-R plasmid that encoded authentic hCKα whose mRNA was not restricted by the hCKα shRNA (TRCN0000284352) was generated by introducing mutations into the shRNA target region of the hCKα open reading frame using a set of mutagenic forward and reverse primers: 5′-GGGGGCTGAGGCCATGGTACTCGAAAGTGTAATGTTTGCCATTCTCGCAG-3′ and 5′-CTGCGAGAATGGCAAACATTACACTTTCGAGTACCATGGCCTCAGCCCCC-3′, respectively. pCMV6-hCKα-D288A-R was generated from pCMV6-hCKα-R using the mutagenic forward and reverse primers 5′-CCAGTTGTATTTTGTCATAATGCCTGTCAAGAAGG-3′ and 5′-CCTTCTTGACAGGCATTATGACAAAATACAACTGG-3′, respectively. The amplicons were subcloned into pCMV6 using SgfI and MluI. Both pCMV6-hCKα-R and pCMV-hCKα-D288A-R were sequenced using T7 primers to confirm the authentic wild-type and D288A mutant hCKα open reading frames.

### *In vitro* RNA transcription, viral genome RNA transfection, and siRNA silencing.

JFH1, JFH1-Luc, and SGR-Luc-JFH1 RNAs were generated as described previously (22, 40, 47). The *in vitro*-transcribed RNAs were electroporated into Huh7 cells based on a previously described procedure (48). For RNA interference (RNAi) silencing studies, 2 × 10^5^ Huh7 cells seeded in six-well plates overnight were transfected with 100 pmol of siRNAs using Lipofectamine RNAi Max (Invitrogen) according to the manufacturer's protocol.

### HCV production, titration, infection, and UV irradiation.

The cell culture-derived infectious HCV (HCVcc) was produced by electroporating JFH1 RNA into Huh7 cells as described above, and then culture supernatants were collected, centrifuged, filtered through 0.45-μm-pore-size cellulose acetate discs, and stored at −80°C. Virus titration was determined as previously described (40, 49). Huh7 cells were infected with HCVcc at a multiplicity of infection (MOI) of 1 according to a previously described procedure (40). At 24 h post-siRNA transfection, cells were left uninfected or infected with HCVcc, and cells were collected at 72 h postinfection for Western blotting, real-time (RT)-PCR, or confocal microscopic analyses. UV inactivation of HCVcc was performed as previously described (50).

### HCV reporter RNA replication and inhibitor assays.

HCV reporter replication was performed using a pipette-type electroporator (Microporator MP-100; Digital Pro), as previously described (40), at 24 h post-siRNA transfection. Transfected cells were harvested at different times after RNA transfection as indicated on the figure, and cell lysates were monitored for firefly luciferase activity. The luciferase activity at 4 h after RNA transfection was used to normalize the transfection efficiency. In inhibitor studies, inhibitors were added at 48 h post-viral RNA transfection and incubated for 24 h before cells were harvested for luciferase assays.

### RT-PCR analysis.

Total RNAs were isolated, reverse-transcribed into cDNAs, and quantified for viral RNA level by RT-PCR using TaqMan Universal master mix II (4440038, Applied Biosystems) and an ABI 7500 real-time PCR system (Applied Biosystems) based on a previously described procedure (40). The quantitative PCR (qPCR) probes used were as follows: Hs99999905 for GAPDH, Hs00957878_m1 for hCKα, Hs00540979_m1 for PEMT, and 6-carboxyfluorescein (6-FAM)-5′-TTCCCGGCAATTCC-3′-minor groove binder and nonfluorescent quencher (MGBNFQ) for HCV. All probes of genes were obtained using TaqMan Gene Expression Assays (Applied Biosystems). The relative amounts of cellular mRNAs and HCV viral RNA were calculated by the comparative threshold cycle (*C_T_*) method (ΔΔ*C_T_*) and normalized to the level of GAPDH. The viral RNA levels in different experimental settings relative to the level detected in mock-infected cells, which was arbitrarily set as 1, were expressed as the fold change in the HCV RNA level. The mRNA levels of cellular genes in knockdown cells relative to the level detected in control knockdown cells, which was arbitrarily set as 1, were expressed as the fold change in the mRNA level.

### Plasmid DNA transfection and coimmunoprecipitation.

Huh7 cells or the paired control and hCKα stable knockdown Huh7 cells were transfected with the plasmids indicated on the figure using Lipofectamine 2000 based on the procedure provided by the manufacturer (Invitrogen). In each transfection analysis, appropriate vector plasmids were added into transfection reaction mixtures to ensure that the total DNA amount in all transfections was identical. In some cases, transfected cells remained mock infected or infected with HCVcc at an MOI of 1 at 48 h post-DNA transfection. Alternatively, transfected cells were transfected with pTM-NS3-NS5B and pWPT-T7-BLR at 48 h post-plasmid transfection. All DNA-transfected cells were harvested at 48 h posttransfection for Western blotting, confocal microscopy, coimmunoprecipitation (co-IP), and transmission electron microscopy (TEM) analyses.

For co-IP analyses, Huh7 or the paired control and hCKα stable knockdown Huh7 cells were cotransfected with the two plasmids indicated on the figure, while HEK293T cells were cotransfected by a standard calcium phosphate coprecipitation method. Cells expressing hCKα were lysed with Tris-HCl buffer containing 1% CHAPS (3-[(3-cholamidopropyl)-dimethylammonio]-1-propanesulfonate). To assess NS protein-protein interactions, transfected cells were lysed with Tris-HCl buffer containing 1% Triton X-100. For co-IP analysis of NS5A produced by the pTM-NS3-NS5B system, Huh7 cells or the control or hCKα stable knockdown Huh7 cells were cotransfected with pWPI-T7-BLR and pTM-NS3-NS5B, and then transfected cells were lysed with Tris-HCl buffer containing 1% CHAPS. For co-IP analysis of NS5A in CK37-treated cells, Huh7 cells stably transduced with the T7 RNA polymerase lentiviral vector (T7 Pol/Huh7) were transfected with pTM-NS3-NS5B. At 24 h post-DNA transfection, cells were treated with or without 100 μM CK37 for 24 h prior to cell harvest. To assess the effect of hCKα activity on the binding of NS5A to hCKα and NS5B, control and hCKα stably knocked down cells that were stably transduced with T7 RNA polymerase lentiviral vector were transfected with wild-type hCKα or hCKα-R or with D288A mutant hCKα-R. At 24 h after transfection, the cells were cotransfected with pTM-NS5A and pTM-NS5B.

For immunoprecipitation, 1 μg of the antibody indicated on the figure was first incubated with protein A Mag Sepharose (28-9670-62; GE Healthcare) at 4°C for 2 h, followed by incubation with cell lysates at 4°C for 6 h. After the beads were washed five times in phosphate-buffered saline (PBS), they were boiled with sample loading buffer and subjected to SDS-PAGE and immunoblot analyses.

### HCV and VSV pseudoparticle package and entry assay.

To produce HCV pseudotyped particles (HCVpp) and vesicular stomatitis virus pseudotyped particles (VSVpp), 293T cells grown in 10-cm dishes were transfected with pNL-Luc-R^−^E^−^ plus pCDNA3-kozakE1E2 (H77 strain), pCX-E1E2-Flag (JFH1 strain), or pMD.G, which encodes the envelope glycoproteins of HCV genotype 1 or 2 or the G protein of vesicular stomatitis virus, using the calcium phosphate coprecipitation method. Two days after transfection, the culture supernatants were harvested, filtered, and used to inoculate siRNA-transfected Huh7 cells for 48 h. The cells were collected 48 h after pseudotyped particle challenge, washed in PBS, and lysed with 1× passive lysis buffer (Promega). Firefly luciferase activity was measured using a Sirus Single Tube Luminometer (Bet-11040010; Berthold Detection Systems). Another set of siRNA-transfected Huh7 cells was harvested at 48 h post-siRNA transfection and analyzed by immunoblotting to assess the knockdown effects of siRNAs transfected.

### Generation of Huh7 cells constitutively expressing control or hCKα shRNA and/or T7 RNA polymerase.

Lentiviral vector stocks encoding control or hCKα shRNAs or T7 RNA polymerase were prepared and transduced into Huh7 cells as previously described (40, 44). The transduced Huh7 cells were selected with 5 μg/ml puromycin (for control and hCKα shRNA/Huh7) or with 10 μg/ml blasticidin (for T7 Pol/Huh7). To obtain Huh7 cells stably expressing T7 RNA polymerase and control or hCKα shRNAs, T7 Pol/Huh7 cells were transduced with lentiviral vectors encoding control or hCKα shRNAs, respectively, and cells were selected with both antibiotics.

### Inhibitor studies and cell viability and PC assays.

All inhibitors were prepared in dimethyl sulfoxide (DMSO) and added to HCVcc-infected or viral RNA-transfected Huh7 cells at 24 h prior to cell harvest. Cells treated with DMSO (vehicle) at the same concentration as that in the inhibitor treatment were used controls. Unless otherwise indicated, the following concentrations of inhibitors were used: CK37, 100 μM; HDTAB, 10 μM; HePC, 10 μM; and AICAR, 250 μM. Cell viability was determined at 2 days post-hCKα siRNA transfection or 24 h after drug treatment using a CellTiter-Glo luminescent cell viability assay kit (G7571; Promega). The amount of PC was measured using a PC assay kit (MAK049; Sigma-Aldrich).

### Membrane flotation, SDS-PAGE, and immunoblot analyses.

Mock- or HCV-infected Huh7 cells were resuspended in PBS containing 0.25 M sucrose (PBS-sucrose) plus a protease inhibitor cocktail (000000004693132001; Roche), and the cells were homogenized using an Omni THQ digital tissue homogenizer. Aliquots containing equal proteins from each postnuclear supernatant were subjected to a 10%/20%/30% discontinuous iodixanol gradient as previously described (51). After the samples were ultracentrifuged, the gradients were fractionated into 22 fractions (500 μl each). The fractions were precipitated with a final concentration of 10% cold trichloroacetic acid prior to Western immunoblotting. SDS-PAGE, Western blotting, and immunoblot visualization were performed as previously indicated (40, 48). The levels of the proteins in the immunoblots were quantified using ImageQuant TL software (GE Healthcare), and the relative protein levels were determined.

### Confocal laser scanning and electron microscopy.

For confocal microscopy, the cells were processed based on previously described procedures (52). Cell samples were mounted with 4′,6′-diamidino-2-phenylindole (DAPI) Fluoromount G (0100-20; Southern Biotechnology Associates), and the specimens were examined using a Zeiss LSM 510 confocal laser scanning microscope (Carl Zeiss, Germany). Images were edited using Zen (version 2011) software. Quantification of colocalization of the proteins was performed using ImageJ or MBF ImageJ software as previously described (53). The cell outline was first drawn by freehand selection, and the region of interest was determined by thresholding each signal intensity. Then, the minimum pixel ratio of channel 1 to channel 2 was set to at least 50% using the colocalization functions provided by the software. In the colocalization images, the percentage of the area that harbored the two-color channels relative to the entire cell area was determined using the measurement function. For three-color fluorescence colocalization, the colocalization image between the first two colors was first generated, and the newly generated image was used as channel 1 to determine colocalization with the third color (as channel 2) as described above.

For TEM analysis, 2 × 10^4^ mock- or HCV-infected cells were reseeded on ACLAR film (50425-10; EMS). At 24 h after the cells were reseeded, the cells on the film were washed three times with PBS and fixed for 30 min in prewarmed 2.5% glutaraldehyde prepared in 50 mM sodium cacodylate buffer (pH 7.2) containing 1 M KCl, 0.1 M MgCl_2_, 0.1 M CaCl_2_, and 2% sucrose. The cells were washed thoroughly five times with 100 mM cacodylate buffer and postfixed in 1% OsO_4_ prepared in 50 mM cacodylate buffer on ice in the dark for 2 h. After the cells were thoroughly washed five times with 50 mM cacodylate buffer, they were successively dehydrated in a graded series of increasing concentrations of ethanol and were finally immersed in LR White resin (62661; Sigma-Aldrich). The embedded cells were sectioned to a thickness of 90 nm using a Leica Ultracut UCT microtome and a diamond knife. The sections were then counterstained with 1% uranyl acetate for 1 h, followed by 2% lead citrate in H_2_O for 5 min, and examined using a JEM-1200EX transmission electron microscope (JEOL USA, Inc.).

### Analyses of data and statistical significance.

The results from RT-PCR, viral RNA replication as determined by luciferase activity, measurement of the PC level, Western blot analysis, and quantification of the relative protein levels in immunoblots were obtained from three independent studies. Western blot data of a representative set are shown. The confocal microscopy and TEM data were obtained from 30 randomly picked cells in three analyses. All data are presented as the means ± standard deviations (SD). Statistical analysis was performed with a two-tailed, unpaired Student's *t* test available in GraphPad Prism software. Statistically significant differences between the two settings shown on the figure are given in the legend.

## RESULTS

### hCKα is required for HCV expression.

To explore whether hCKα plays a role in HCV infection, we conducted siRNA-based hCKα silencing and assessed its effect on HCV expression. Transfection of hCKα siRNA into HCV-infected cells effectively knocked down hCKα expression (Fig. 1B). This knockdown of hCKα expression was concomitant with the reduced expression of HCV proteins, such as NS3, NS5A, and core proteins, compared to that detected in control siRNA-transfected cells (Fig. 1B). Of note, HCV infection did not obviously affect the level of hCKα expression (Fig. 1B, compare lanes 1 and 2). In parallel, transfection of hCKα siRNA into HCV-infected cells not only reduced the hCKα mRNA level (data not shown) but also profoundly diminished the HCV RNA level (Fig. 1C).

To determine whether hCKα is required for cell growth under our experimental conditions, Huh7 cells transfected with control or hCKα siRNA were assessed for cell viability. Cells transfected with hCKα siRNA exhibited cell viability comparable to that of cells transfected with control siRNA (Fig. 1D, left panel). Thus, the decreased expression of viral RNA and proteins in hCKα siRNA-treated cells (Fig. 1B and C) was not due to growth retardation caused by hCKα knockdown but, rather, to the specific effect of hCKα depletion on HCV expression.

### hCKα promotes HCV protein expression.

To rule out the nontargeted effect of hCKα siRNA on inhibited viral protein expression, we determined whether this kinase could directly promote HCV replication by performing a rescue assay. We first generated untargeted control and hCKα stable knockdown Huh7 cells by lentiviral transduction. Immunoblot analysis revealed that the hCKα protein level was markedly lower in the cells expressing the hCKα shRNA than that detected in cells expressing control shRNA (Fig. 1E). These paired stable knockdown Huh7 cells also showed comparable cell viability (Fig. 1D, right panel). We then constructed an hCKα-R plasmid that encoded authentic hCKα whose mRNA was insensitive to the restriction effect caused by the hCKα-specific shRNA used to construct the hCKα stable knockdown cells. In the HCV-infected control shRNA stable cells, overexpression of both normal hCKα and hCKα-R increased NS3, NS5A, and core protein expression compared with levels in infected cells without hCKα overexpression (Fig. 1E, upper panel). Additionally, overexpression of hCKα or hCKα-R in HCV-infected cells stably expressing hCKα shRNA partially rescued HCV expression (Fig. 1E, upper panel). Protein quantitation showed that hCKα or hCKα-R overexpression increased the NS3, NS5A, and core protein levels in both control and hCKα stable knockdown cells (Fig. 1E, lower panel). These results collectively demonstrate that hCKα positively modulates HCV protein expression.

### The enzymatic activity of hCKα is required for HCV RNA replication.

**FIG 2:**
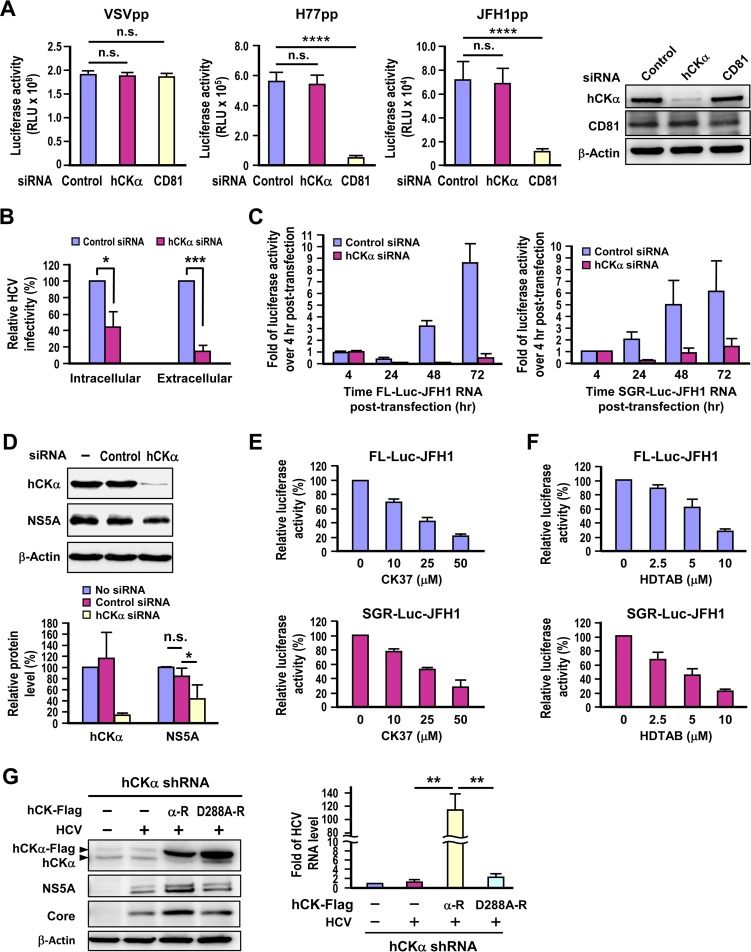
Requirement of hCKα for HCV RNA replication. (A) Huh7 cells transfected with control or hCKα- or CD81-specific siRNAs for 48 h were separately infected with HCVpp generated from the genotype 1a H77 strain and genotype 2a JFH1 strain or with VSVpp, and luciferase activities were measured. In parallel, a set of cells harvested at 48 h post-siRNA transfection was analyzed by Western blotting; a representative data set is shown. (B) Huh7 cells transfected with control or hCKα-specific siRNAs were infected with HCV, and the infectivities of the extracellular and intracellular viruses were assessed. This analysis was performed by inoculating culture medium that contained extracellular virions and cell extracts that contained intracellular virions, respectively, into naive Huh7.5-1 cells. The infected cells were harvested, and isolated total RNA was analyzed for the HCV RNA level by RT-PCR. (C) Huh7 cells were transfected with FL-Luc-JFH1 RNA or SGR-Luc-JFH1 RNA, as indicated. HCV RNA replication was measured by determining the fold change in firefly luciferase activity at different time points over that determined at 4 h after RNA transfection. (D) Huh7 cells stably replicating genotype 1b Con1 strain SGR remained untransfected or were transfected with the indicated siRNAs, and cell lysates were analyzed by Western blotting (top panel). The level of the indicated proteins in different settings relative to that detected in siRNA-untreated SGR-replicating cells, which was arbitrarily designated 100%, was expressed as indicated (bottom panel). (E and F) FL-Luc-JFH1 or SGR-Luc-JFH1 RNA-transfected cells were treated with different concentrations of CK37 or HDTAB, as indicated, and the luciferase activity was measured and expressed as the percentage of that detected in cells that received vehicle treatment. (G) Huh7 cells constitutively expressing hCKα shRNA were transfected with control vector (−) or wild-type (α-R) or D288A (D288A-R) hCKα resistant to hCKα shRNA, and cells were left uninfected or infected with HCV. Duplicate sets of cells were analyzed by Western blotting for protein expression (left panel) and by RT-PCR for the viral RNA level (right panel). *, *P* < 0.05; **, *P* < 0.01; ***, *P* < 0.001; ****, *P* < 0.0001; ns, nonsignificant.

Next, we determined whether hCKα participated in HCV entry into host cells using the HCV pseudotyped particle (HCVpp) entry assay (54). As a positive control for the inhibitory effect of siRNA transfection on viral entry, transfection with siRNA specific for CD81, a critical viral entry coreceptor (55, 56), was used as a control. Transfection with CD81 siRNA reduced the CD81 protein by 52% compared to that detected in control siRNA-transfected cells (Fig. 2A). This partial reduction in the CD81 level produced a significant inhibitory effect on the entry of genotype 1a H77 strain and genotype 2a JFH1 strain HCVpp, but not that of VSVpp (Fig. 2A). In contrast, transfection with hCKα siRNA reduced the hCKα protein by 90% but neither affected the entry of HCVpp derived from both genotypes nor altered the entry of VSVpp into Huh7 cells (Fig. 2A). These observations excluded possible effects of hCKα on HCV entry. We also compared the infectivity of intracellular and extracellular viruses obtained from control and hCKα-silenced cells infected with HCV by inoculating the viruses into naive Huh7 cells, and the total RNA isolated was quantified to determine the viral RNA level by RT-PCR. hCKα knockdown reduced both the intracellular and extracellular viral infectivity in parallel (Fig. 2B), indicating that this kinase does not play an obvious role in the assembly or release of infectious HCV particles.

To assess the contribution of hCKα to HCV RNA replication, we studied the effect of hCKα knockdown on the HCV RNA replication kinetics of a bicistronic, full-length (FL) Luc-JFH1 genome that also encodes a firefly luciferase gene as a reporter (57). In the untargeted knockdown cells, luciferase activity decreased from 4 to 24 h post-viral RNA transfection and then gradually increased with time (Fig. 2C, left panel), exhibiting a lag between translation of the initial incoming viral genome before active RNA replication. Nevertheless, luciferase activity was greatly reduced at different time points post-viral RNA transfection in the hCKα knockdown cells in contrast to their control knockdown counterparts (Fig. 2C, left panel). Likewise, the replication kinetics of a subgenomic replicon (SGR) Luc-JFH1 (43) were also substantially attenuated in the hCKα knockdown cells compared to those detected in the control knockdown cells (Fig. 2C, right panel). Furthermore, silencing of hCKα in Huh7 cells stably expressing the genotype 1b Con1 SGR, which did not produce infectious virus particles, also curtailed NS5A expression (Fig. 2D), indicating that transfection with hCKα siRNA still confers an inhibitory effect on HCV RNA replication although the virus has established viral replication in host cells. Taken together, these observations indicate that the inhibitory effect of hCKα knockdown on viral RNA replication is not dependent on the entire virus life cycle or on the virus genotype examined.

To test whether hCKα activity is required for HCV RNA replication, the effect of CK37, a specific inhibitor of hCKα enzymatic activity (36), on viral RNA replication was examined. The viral RNA replication of FL and SGR Luc-JFH1 was inhibited by CK37 in a dose-dependent manner (Fig. 2E). Similarly, treatment with HDTAB, another hCKα activity inhibitor (58), also interfered with viral RNA replication of the FL and SGR Luc-JFH1 genomes proportional to the concentration of HDTAB used (Fig. 2F).

Generally, eight highly conserved domains exist among CKs from different species, such as rat, mouse, Drosophila, Saccharomyces cerevisiae, and human (59). The main catalytic sites of CK have been reported to be located in domains 6 and 7. Domain 6, also known as the Brenner phosphotransferase motif, has the conserved sequence HXDhXXXNhhh…..D, in which “X” stands for any amino acid, “h” stands for large hydrophobic amino acids (such as F, L, I, M, V, W, or Y), and the ellipses represent the variable lengths of amino acids in different species (59). Residues in the Brenner phosphotransferase motif in CK participate in maintaining the structure of the ATP binding site and the orientation of choline (60). The negative charge of Asp-306 in hCKα2, equivalent to Asp-288 in hCKα1, deprotonates the hydroxyl group of the ATP substrate, maintains the proper active site, and increases the electrophilicity of ATP γ-phosphate through hydrogen bonding (61). In CKs from other species, mutations introduced into the Asp residue analogous to the mutation in hCKα1 Asp-288, for instance, mutation of Asp-255 in Caenorhabditis elegans CK to Ala or Asn, impair the enzymatic activity (62).

To confirm that hCKα activity is essential for viral replication, we examined the ability of the Asp-to-Ala mutation introduced at residue 288 of the hCKα active site to rescue HCV replication in hCKα stable knockdown cells. Toward this end, we constructed a D288A-R mutant based on the backbone of hCKα-R. Unlike overexpression of the wild-type hCKα-R in hCKα stable knockdown cells, which substantially enhanced viral protein expression, overexpression of the D288A-R mutant did not effectively increase HCV protein expression despite comparable expression of wild-type and mutant hCKα-R proteins (Fig. 2G, left panel). In accordance, overexpression of wild-type hCKα-R, not the D288A-R mutant, upregulated the viral RNA level in the HCV-infected hCKα stable knockdown cells (Fig. 2G, right panel). These findings clearly indicate that the enzymatic activity of hCKα is required for upregulating HCV RNA replication.

### The CDP-choline pathway is not essential for HCV replication.

**FIG 3:**
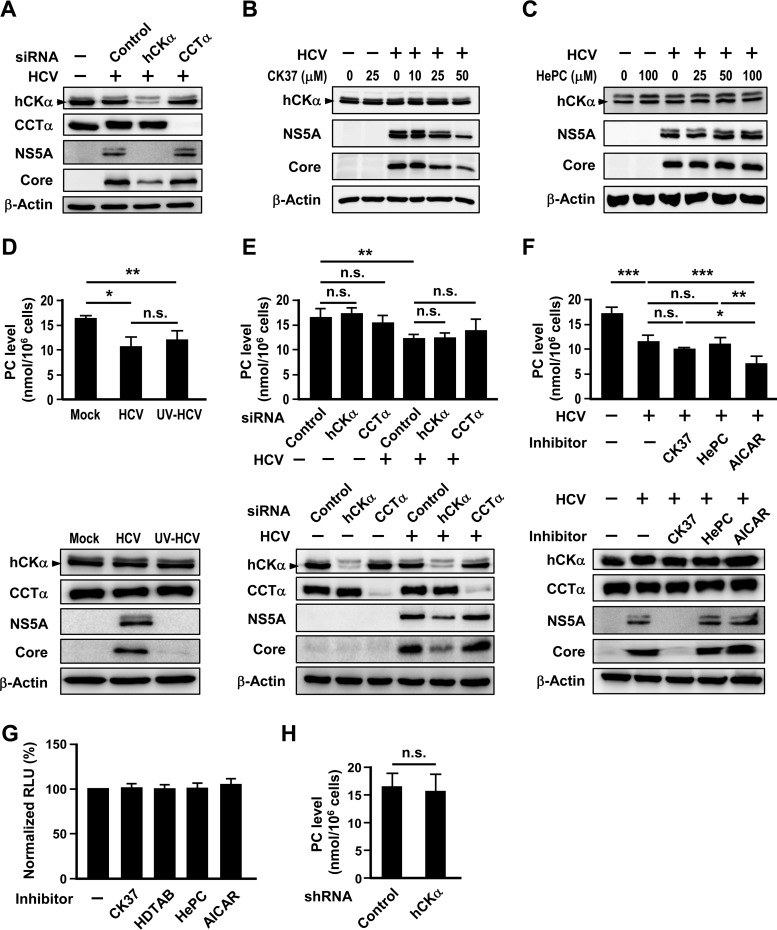
No apparent role of the CDP-choline pathway in HCV replication. (A) Huh7 cells remained untransfected or were transfected with the indicated siRNAs and then mock infected or infected with HCV. Cell lysates were subjected to immunoblot analysis. (B and C) Huh7 cells were mock infected or infected with HCV and then treated with different concentrations of CK37 or HePC, as indicated. Cells lysates were analyzed by immunoblotting. (D) Huh7 cells remained mock infected or were inoculated with untreated or UV-irradiated HCV. (E) Huh7 cells were first transfected with the indicated siRNAs and then mock infected or infected with HCV. (F) Huh7 cells were mock infected or infected with HCV and then treated with DMSO (−), CK37, HePC, or AICAR, as indicated, for 24 h. For the experiments shown in panels D to E, a set of 10^6^ cells was used to determine the PC amount (top panel), while another set was analyzed by Western blotting (bottom panel). (G) The viability of Huh7 cells treated with vehicle or the indicated inhibitors was assessed. (H) The PC levels in control and hCKα stable knockdown cells were measured. *, *P* < 0.05; **, *P* < 0.01; ***, *P* < 0.001; ns, nonsignificant.

To understand the specificity of hCKα for HCV replication, the effect of knocking down CCT isoform α (CCTα), the rate-limiting enzyme in the CDP-choline pathway, on HCV expression was studied. In contrast to the effect of hCKα knockdown on reduced core and NS5A protein expression, CCTα knockdown did not affect HCV expression (Fig. 3A). Additionally, in contrast to the dose-dependent inhibitory effect of CK37 on viral protein expression (Fig. 3B), up to 100 μM HePC, which was shown to inhibit PC biosynthesis in HepG2 cells through inhibiting CCT activity (63), did not apparently affect HCV expression in Huh7 cells (Fig. 3C). These results emphasize that hCKα activity is specifically required for HCV replication.

We then determined whether HCV infection might affect the PC level. Unexpectedly, HCV infection decreased the intracellular PC level (Fig. 3D). Huh7 cells inoculated with UV-irradiated HCV particles also reduced the PC level (Fig. 3D). Moreover, we found that neither silencing of hCKα nor knockdown of CCTα in mock-infected cells or HCV-infected cells affected the PC level (Fig. 3E). Consistent with this observation, treatment with CK37 or HePC did not affect the PC level in the HCV-infected cells (Fig. 3F). As a positive control for the altered PC level in this assay, treatment of HCV-infected cells with AICAR, a commonly used indirect activator of AMP-activated protein kinase (64) that is known to attenuate PC production in freshly isolated mouse hepatocytes (65), decreased the PC level in infected cells compared to that in infected cells treated with vehicle (Fig. 3F, top panel). Nonetheless, the AICAR-induced reduction in the PC level did not affect HCV expression (Fig. 3F, bottom). Of note, treatment of Huh7 cells with CK37, HDTAB, HePC, or AICAR at the concentrations indicated in Materials and Methods did not noticeably affect cell viability (Fig. 3G). Additionally, the control and hCKα stable knockdown cells expressed comparable levels of PC (Fig. 3H), consistent with the comparable cell viability observed with the paired control and hCKα stable knockdown cells (Fig. 1D, right panel). Collectively, these results indicate that Huh7 cells with transient knockdown or inactivation of hCKα or CCTα or cells with constitutive hCKα knockdown still preserve PC biosynthesis and that the PC level does not directly influence viral replication. Thus, we conclude that hCKα activity, not the CDP-choline pathway, is required for HCV replication.

### Impairment of the CDP-choline pathway upregulates PEMT expression.

**FIG 4:**
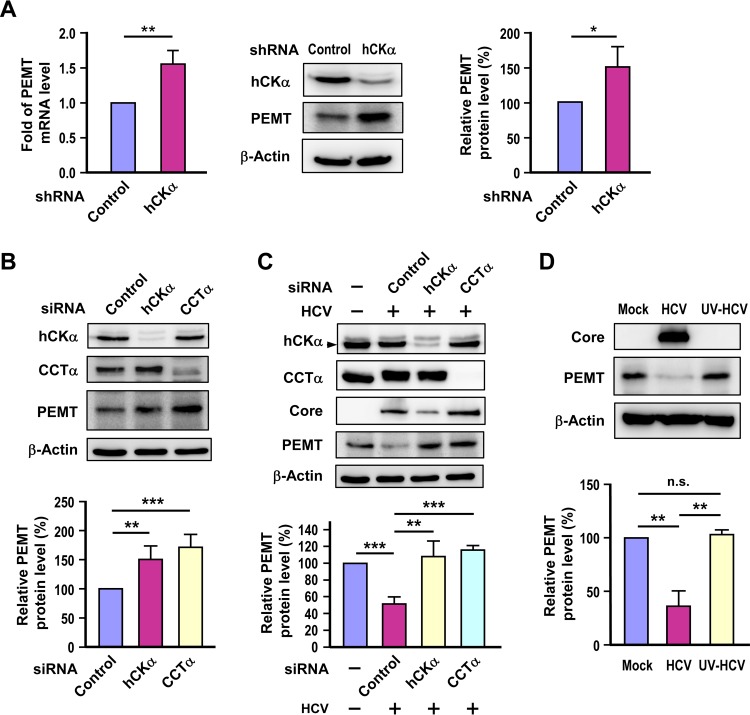
Effect of disruption of the CDP-choline pathway on PEMT expression. (A) PEMT mRNA and protein expression levels were determined in control and hCKα stable knockdown cells by RT-PCR (left panel) and Western blot (middle and right panels) analyses, respectively. (B) Huh7 cells were transfected with the indicated siRNAs, and cell lysates were subjected to immunoblot analysis to determine the expression of the indicated proteins. (C) Huh7 cells were transfected with the indicated siRNAs and then mock infected or infected with HCV. Cell lysates were then analyzed by Western blotting. (D) Huh7 cells were mock infected or challenged with untreated HCV or UV-irradiated HCV, and then cell lysates were subjected to immunoblot analysis. *, *P* < 0.05; **, *P* < 0.01; ***, *P* < 0.001; ns, nonsignificant.

As the PC level was preserved despite impairment of the CDP-choline pathway by hCKα or CCTα knockdown (Fig. 3E, F, and H), we then examined whether the alternative route that synthesizes PC from PE by the PEMT pathway (Fig. 1A) was induced when the CDP-choline pathway was disrupted in Huh7 cells. RT-PCR and Western blot analyses showed that the PEMT mRNA and protein levels were higher in the hCKα stable knockdown cells than in the control stable knockdown cells (Fig. 4A).

Next, we determined whether hCKα or CCTα transient knockdown in uninfected cells might upregulate PEMT expression. A higher level of PEMT protein was detected in hCKα or CCTα transient knockdown cells than that detected in control knockdown cells (Fig. 4B). We then assessed whether depletion of hCKα or CCTα in the HCV-infected cells also induced PEMT protein expression. To our surprise, HCV infection resulted in a decrease in PEMT protein expression compared to the level in mock infection (Fig. 4C). Notably, silencing of hCKα or CCTα restored PEMT protein expression back to the same level as that detected in mock-infected cells (Fig. 4C). To examine whether the reduced PEMT expression in HCV-infected cells was specific to HCV infection, PEMT protein levels were compared in cells inoculated with untreated or UV-irradiated HCV. The PEMT protein level was decreased in HCV-infected cells but not in cells challenged with UV-irradiated HCV (Fig. 4D). Taken together, these results demonstrate that the PEMT pathway is activated in response to impairment of the CDP-choline pathway in both uninfected and HCV-infected Huh7 cells.

### HCV infection increases hCKα localization on the ER.

**FIG 5:**
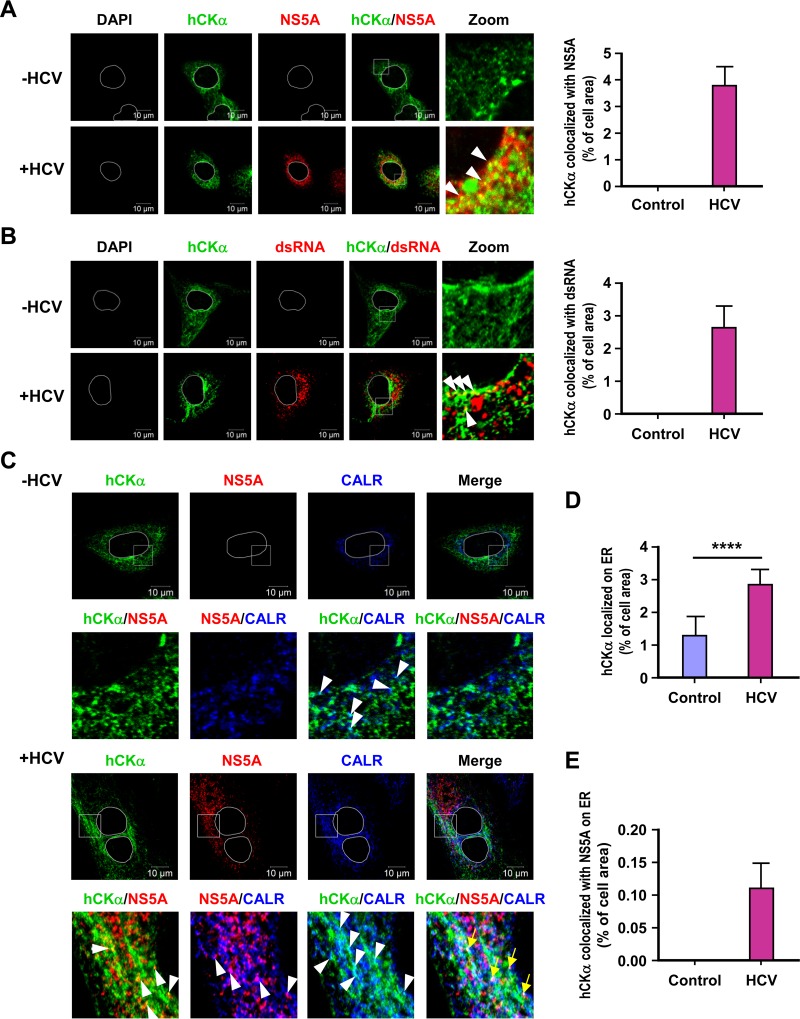
Intracellular localization of hCKα and the hCKα-associated viral RC on the ER. (A and B) Mock- and HCV-infected Huh7 cells were fixed and processed by confocal microscopy to examine the intracellular localization of hCKα along with NS5A (A) or dsRNA (B), and the two fluorescence profiles were merged (left panels). The boxed areas in the hCKα/NS5A and hCKα/dsRNA images were enlarged (Zoom) to show colocalization (white arrowheads) of hCKα with NS5A or dsRNA. The degree of colocalization of hCKα with NS5A or dsRNA was calculated as the percentage of the cell area that harbored the indicated fluorescence signal relative to the entire cell area, as described in Materials and Methods (right panels). (C to E) Mock- and HCV-infected Huh7 cells were examined by confocal microscopy to determine the intracellular localization of the indicated proteins (C). Merged images show the overlay of the three fluorescence signals. The second row of images shows enlargements of the boxed areas in the top row. The white arrowheads indicate the colocalization of the two indicated fluorescence signals, whereas the yellow arrows indicate the colocalization of the three fluorescent signals. The degrees of colocalization of hCKα and CALR and of hCKα, NS5, and CALR were quantified (D and E, respectively). ****, *P* < 0.0001.

In accordance with the indispensable role of hCKα in HCV RNA replication, confocal microscopy showed that a small fraction of the hCKα molecules colocalized with NS5A in HCV-infected cells (Fig. 5A, left panel). Likewise, in HCV-infected cells, a small portion of hCKα could colocalize with double-stranded RNA (dsRNA), which is used to denote the nascent viral dsRNA intermediate newly synthesized during viral replication (Fig. 5B). The percentage of the colocalization area of hCKα and NS5A or dsRNA relative to the entire cell area, which was calculated as previously indicated (53), is shown in the right panels of Fig. 5A and B, respectively.

Because the HCV polyprotein precursor is synthesized and proteolytically processed on the ER membrane and because the ER-derived altered membrane serves as a critical organelle that houses the viral replication factories (66, 67), we sought to examine whether HCV infection affected the intracellular localization of hCKα to the ER using an antibody against CALR, an ER marker. We noted that a small fraction of hCKα localized on the ER, as shown by the presence of cyan dots in both mock- and HCV-infected cells (Fig. 5C). Nevertheless, the HCV-infected cells exhibited a greater number of cyan puncta than the mock-infected cells (Fig. 5C). In accordance, quantification analysis showed that HCV infection increased the colocalization of hCKα with CALR compared to that with mock infection (Fig. 5D). We also noted that a small portion of viral RCs bearing both hCKα and NS5A signals was detected on the ER, as judged by the appearance of white puncta in infected cells (Fig. 5C, hCKα/NS5A/CALR, yellow arrows), and approximately 0.11% of the cell area harbored all three fluorescence signals (Fig. 5E).

### HCV infection increases the cofractionation of hCKα with viral proteins in the ER membrane.

**FIG 6:**
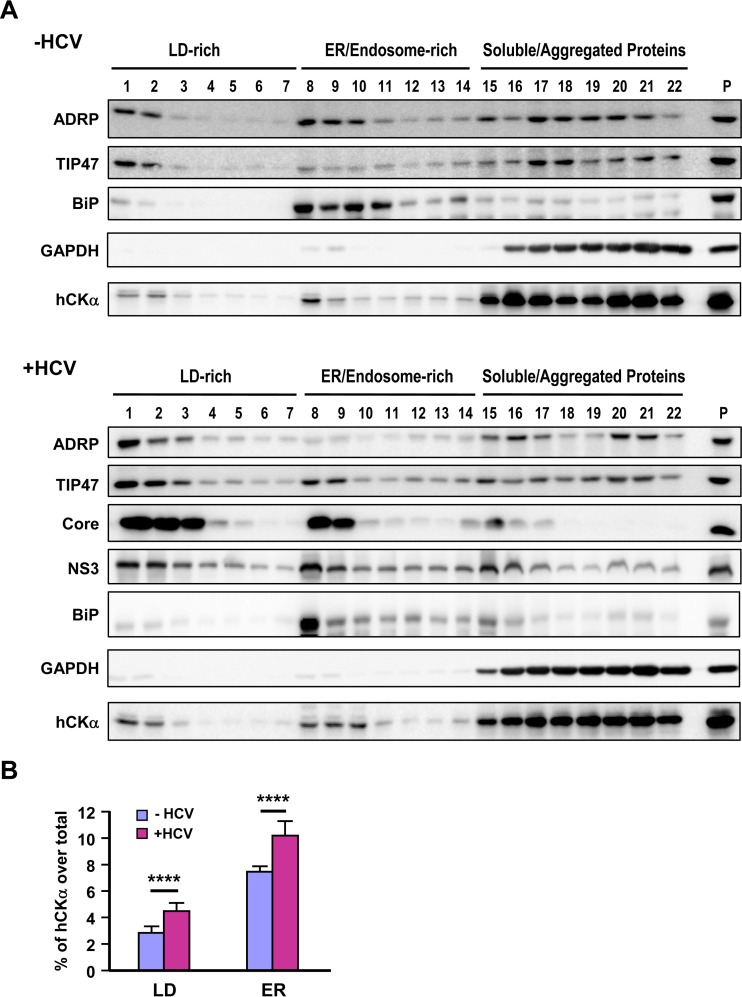
HCV-induced increase in the distribution of hCKα into ER membranes by HCV infection. (A) The postnuclear fractions from mock- and HCV-infected Huh7 cells were subjected to iodixanol-based OptiPrep density gradient ultracentrifugation. Proteins in each fraction were precipitated with 10% cold trichloroacetic acid and then analyzed by Western blotting for the indicated proteins. Extracts from mock- and HCV-infected cell lysates were separately loaded as controls for the detection of the indicated proteins (P lanes). (B) The intensity of hCKα in each fraction was quantified by ImageQuant, and the percentage of hCKα fractionated into LDs (fractions 1 to 7) and the ER (fractions 8 to 14) over the total hCKα level obtained from mock- and HCV-infected cells was quantified. ****, *P* < 0.0001.

To provide biochemical evidence for the association of hCKα with the ER, we performed iodixanol-based density gradient subcellular fractionation (51). In the context of mock infection or HCV infection, BiP, a chaperone located in the lumen of the ER, and GAPDH, a cytosolic protein, were predominantly fractionated into fractions 8 and 9 and fractions 15 to 22, representing the ER/endosome and soluble/aggregated protein fractions, respectively (Fig. 6A). The top two light-density fractions included the LD-associated membranes, as the two LD markers ADRP and TIP47 were distributed in these fractions (Fig. 6A). Quantification analyses from three independent studies showed that HCV infection increased the distribution of ADRP and TIP47 in LD-rich membranes (*P* < 0.05; data not shown). In this regard, Vogt et al. showed that HCV infection or viral RNA replication augments the distribution of TIP47, but not ADRP, in the LD fractions and that TIP47 interacts with NS5A and recruits LD-rich membranes to the MW for efficient viral RNA replication (51). In HCV-infected cells, a fraction of hCKα cofractionated with the HCV core and NS3 proteins, which are recruited to LD-rich compartments during HCV viral assembly (Fig. 6A, bottom panel). Furthermore, hCKα also cofractionated with core and NS3 proteins into the ER/endosome-rich membrane fractions (Fig. 6A, bottom panel). As NS3 is a component of the HCV RC, these findings support a model in which hCKα associates with the HCV RC on the ER and LD-rich membranes. Quantitative analysis revealed that HCV infection increased hCKα distribution into LD and ER fractions (Fig. 6B), suggesting that hCKα enrichment in these two compartments is crucial for viral replication.

### Knockdown or inactivation of hCKα abrogates HCV-induced MW formation.

Because hCKα is a critical regulator of HCV RNA replication (Fig. 1 and 2) and because MW is critical for viral RNA replication (38, 68, 69), we then examined whether hCKα participates in MW formation by employing the pTM transient expression system, which was shown to mediate formation of the viral RC complex and MW independent of viral RNA replication (44, 70, 71).

**FIG 7:**
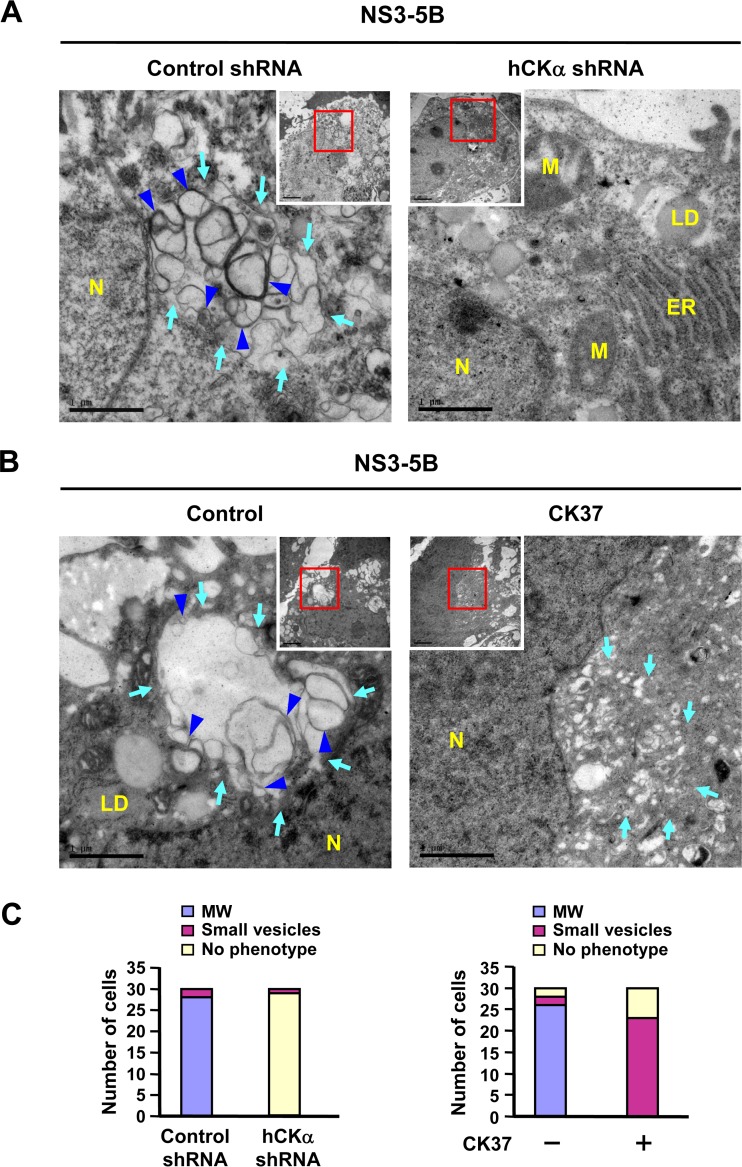
Abrogation of HCV-induced MW formation by hCKα depletion or CK37 treatment. (A) Control shRNA and hCKα shRNA stable expression cells were transfected with pTM-NS3-5B and pWPI-T7-BLR, fixed, and processed for TEM. (B) Huh7 cells were first cotransfected with plasmids expressing T7 polymerase and NS3-NS5B. At 24 h post-DNA transfection, cells were left untreated or treated with 100 μM CK37 for 18 h, and then cells were analyzed by TEM for MW formation. The area boxed in red was enlarged as shown. Light blue arrows indicate vesicles of heterogeneous sizes (left panels of A and B) and small vesicles in CK37-treated Huh7 cells (right panel of B); blue arrowheads indicate double-membrane vesicles (DMVs) (left panels of A and B). N, nucleus; LD, lipid droplet; ER, endoplasmic reticulum; M, mitochondria. (C) Thirty cells randomly picked from each experimental setting were examined by TEM, and the numbers of cells showing three different phenotypes were quantified. MW, vesicles 130 to 270 nm in diameter; small vesicles, vesicles 40 to 100 nm in diameter.

TEM analysis revealed that expression of NS3 to NS5B in the control shRNA knockdown Huh7 cells resulted in significant MW formation, as indicated by the presence of membrane matrix in the perinuclear region, inside which vesicles of heterogeneous sizes between 130 and 270 nm in diameter were enclosed (Fig. 7A, left panel, light blue arrows). Among these vesicles, double-membraned vesicles (DMVs) were noted (Fig. 7A, left panel, blue arrowheads). These DMV structures were shown to be viral replication factories because the kinetics of their appearance correlated with the viral RNA replication rate (9) and because purified DMVs contained HCV replicase proteins such as NS3 and NS5A (10). Small round vesicles with a diameter ranging from 40 to 100 nm were sometimes observed scattered around the perinuclear region. In the hCKα stable knockdown cells, intact ER and mitochondria were evident, and no typical MW structures were observed (Fig. 7A, right panel). The quantitative results regarding MW formation, small-vesicle formation, or absence of phenotype for 30 randomly picked cells is summarized in the left panel of Fig. 7C.

To understand whether hCKα activity is essential for MW formation, Huh7 cells expressing NS3-NS5B were treated with or without CK37. Unlike the vesicles enclosed within the MW structure (Fig. 7B, left panel), no typical MW structure was observed in CK37-treated cells. Instead, small vesicles, sometimes with an elongated shape, were observed scattered around the perinuclear region (Fig. 7B, right panel, light blue arrows). These vesicles were smaller than those vesicles enclosed within the MW structure. Additionally, a portion of CK37-treated cells exhibited no MW or small-vesicle phenotype. The right panel of Fig. 7C summarizes the quantitative results regarding the three phenotypes observed for cells expressing NS3-NS5B that were treated with vehicle or CK37. These observations demonstrate a correlation between the inhibitory effects of CK37 on MW formation and viral RNA replication, implying that hCKα activity contributes to MW formation.

### hCKα is recruited onto the viral RC, presumably through its interaction with D1 of NS5A.

**FIG 8:**
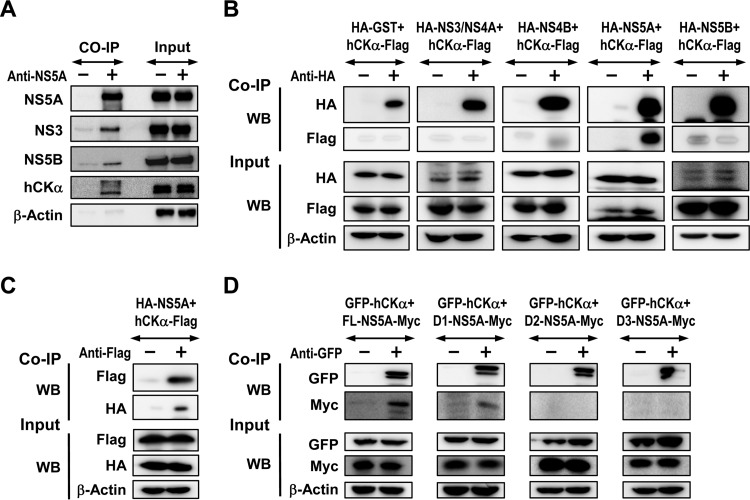
Assessment of the interaction of hCKα with NS5A. (A) Huh7 cells coexpressing T7 RNA polymerase and NS3-NS5B were lysed and subjected to co-IP with an anti-NS5 MAb or an isotype-matched control mouse IgG. The precipitates were analyzed by SDS-PAGE, followed by immunoblotting analysis to detect each of the indicated proteins. A portion of aliquots containing 5% of the total proteins in the lysates used for precipitation was loaded as the input control. (B) 293T cells were cotransfected with plasmids encoding each of HCV genotype 1b Con1 strain NS proteins, as indicated, with a pCMV6 plasmid encoding hCKα-Flag. Cell lysates were pulled down with rabbit anti-HA, and the precipitated proteins were subjected to immunoblot analysis with the indicated MAb antibodies. (C) Lysates from Huh7 cells expressing the indicated proteins were precipitated with rabbit anti-Flag, and the coprecipitated proteins were subjected to immunoblot detection with the indicated MAbs. (D) Lysates from Huh7 cells coexpressing GFP-tagged hCKα and different Myc-tagged NS5A domains were pulled down with rabbit anti-GFP, followed by immunoblot analysis using the indicated MAbs.

To understand the function of hCKα in viral RC and MW formation, we performed co-IP assays to examine whether hCKα was recruited to the viral RC that formed in the context of the pTM expression system. Precipitation of lysates expressing proteins NS3 to NS5B with an NS5A MAb not only precipitated NS5A but also specifically cocaptured NS3 and NS5B (Fig. 8A), indicating viral RC formation. In addition, hCKα was coprecipitated with the viral RC using NS5A MAb but not using control IgG (Fig. 8A), showing that hCKα is recruited to the viral RC.

To determine the mechanism by which hCKα is recruited by the viral RC, we studied which NS protein interacts with hCKα by co-IP. Lysates from cells coexpressing a series of HA-tagged NS proteins derived from the genotype 1b Con1 strain and Flag-tagged hCKα were precipitated with rabbit anti-HA, and the precipitated proteins were analyzed by Western blotting. We found that hCKα-Flag was predominantly associated with NS5A but not with other NS proteins (Fig. 8B). Conversely, precipitating hCKα-Flag with anti-Flag also pulled down HA-NS5A (Fig. 8C). Domain mapping using GFP-hCKα and a set of constructs encoding different domains of the JFH1 strain NS5A with a Myc tag at their C termini (45) showed that D1 of NS5A mediated the NS5A-hCKα interaction, as anti-GFP coprecipitated FL and D1, but not D2 and D3, of NS5A (Fig. 8D). The results also demonstrated that hCKα interacts with NS5A in a genotype-independent manner.

### hCKα activity mediates the enhanced colocalization of hCKα and NS5A on the ER.

**FIG 9:**
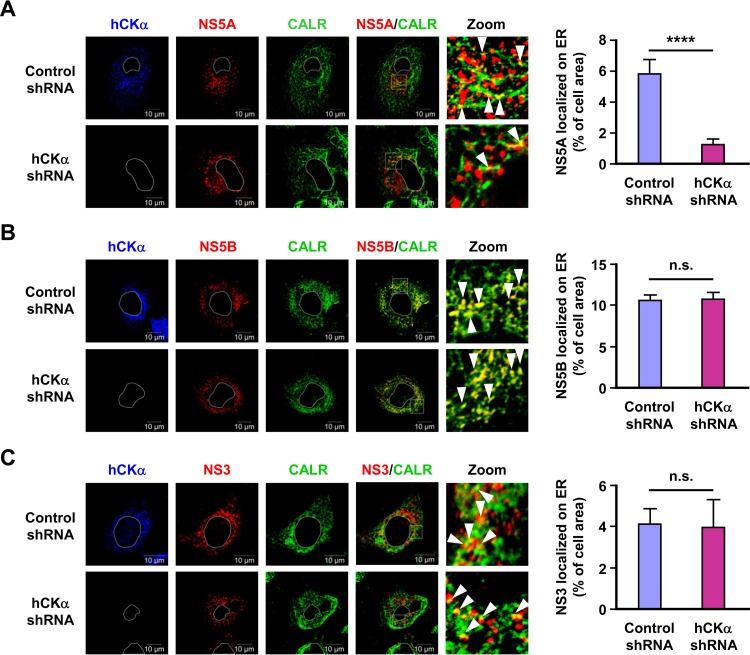
The effect of hCKα knockdown on ER localization of NS5A, NS5B, and NS3. The control and hCKα shRNA Huh7 stable cells were transfected with pTM-NS3-5B and pWPI-T7-BLR, and the cells were examined by confocal microscopy for ER localization of NS5A (A), NS5B (B), and NS3 (C) (left panels). The white boxed areas in the NS/CALR images were enlarged (Zoom), and white arrowheads indicate the ER localization of each indicated NS protein. The extent of colocalization of each NS protein with CALR in control and hCKα shRNA cells was quantified (right panel). ****, *P* < 0.0001; ns, nonsignificant.

As hCKα is a critical NS5A-interacting partner, we then assessed whether hCKα contributed to the localization of NS5A on the ER. hCKα stable knockdown cells showed decreased localization of NS5A on the ER compared with the level of the control stable knockdown cells (Fig. 9A). In contrast, stable hCKα knockdown did not affect the localization of NS3 or NS5B on the ER (Fig. 9B and C, respectively). These results indicate that hCKα enhances the localization of NS5A to the ER and suggest that hCKα may stabilize the association of NS5A with other NS proteins.

**FIG 10:**
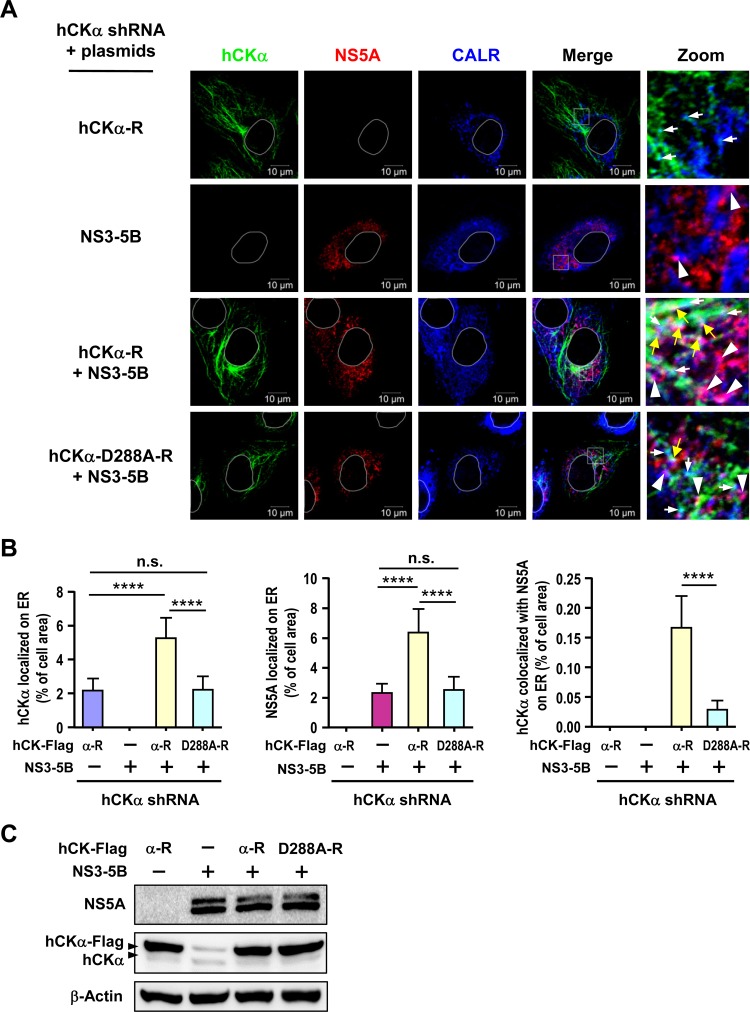
Analysis of the effect of hCKα activity on ER localization of hCKα and NS5A. (A) The hCKα stable knockdown Huh7 cells were cotransfected with pWPI-T7-BLR along with wild-type or D288A mutant hCKα-R plasmid in the presence or absence of pTM-NS3-NS5B as indicated. The cells were then analyzed by confocal microscopy for the intracellular localization of hCKα and NS5A on the ER. The white boxed areas in merged images were enlarged (Zoom) to show the localization of hCKα and NS5A on the ER (white arrows and white arrowheads, respectively) and the colocalization of hCKα and NS5A on the ER (yellow arrows). (B) The degree of localization of hCKα, NS5A, and hCKα-associated NS5A on the ER was quantified. (C) A representative set of Western blot results for the indicated proteins is shown. ****, *P* < 0.0001; ns, nonsignificant.

To explore the mechanism by which hCKα targets NS5A to the ER, we examined whether hCKα activity affected the ER localization of NS5A and hCKα and whether the expression of NS proteins enhanced hCKα localization to the ER. To this end, we examined the intracellular localization of NS5A and hCKα in hCKα stable knockdown Huh7 cells overexpressing wild-type or D288A mutant hCKα-R, with or without NS3-NS5B expression, by confocal microscopy (Fig. 10A). A basal level of overexpressed wild-type hCKα was localized on the ER in the absence of NS3-NS5B expression (Fig. 10B, left panel). Expression of NS3-NS5B augmented ER localization of wild-type hCKα but not of overexpressed D288A mutant hCKα (Fig. 10B, left panel). This observation was reminiscent of the earlier observation that HCV infection increased hCKα localization on the ER (Fig. 5C and D). In contrast, a basal level of NS5A was detected on the ER in the absence of wild-type hCKα expression (Fig. 10B, middle panel). Interestingly, overexpression of wild-type, but not D288A mutant, hCKα-R resulted in greater accumulation of NS5A on the ER (Fig. 10B, middle panel). Moreover, overexpression of wild-type hCKα enhanced hCKα colocalization with NS5A on the ER; however, overexpression of the D288A mutant resulted in the colocalization of only a basal level of hCKα with NS5A on the ER (Fig. 10B, right panel). These results collectively indicate that NS3-NS5B expression promotes hCKα trafficking to the ER and that hCKα activity is required for effective targeting of NS5A, hCKα, and the hCKα-NS5A complex to the ER.

### hCKα is necessary for the interaction of NS5A with NS5B in the viral RC.

**FIG 11:**
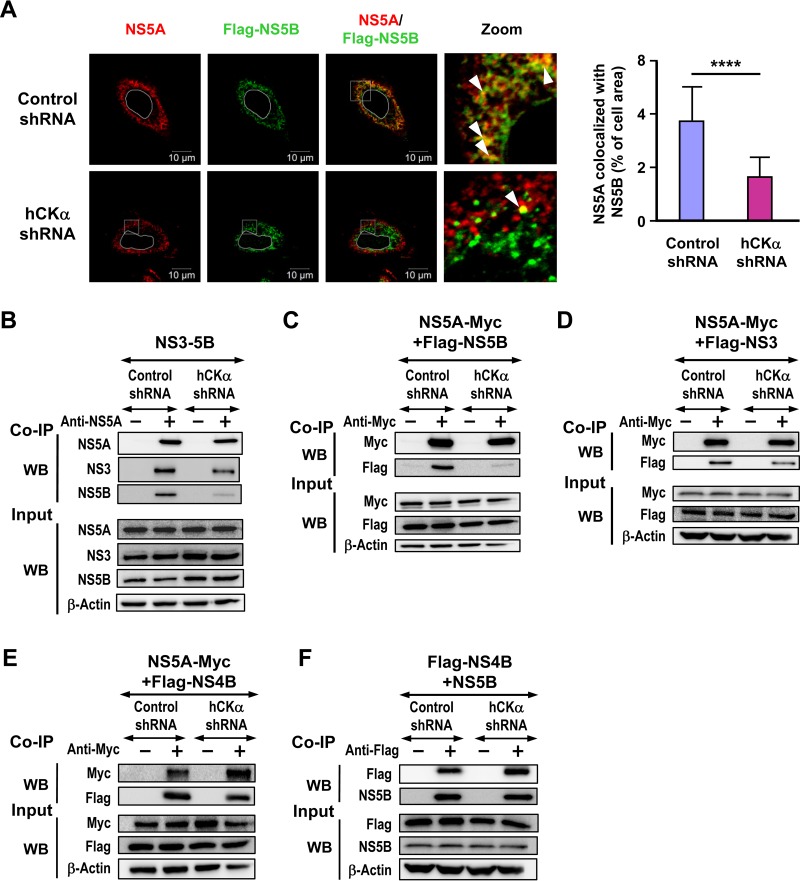
Requirement of hCKα for the binding of NS5A to NS5B in the viral RC. (A) Huh7 cells were cotransfected with pTM-NS3-5B and pWPI-T7-BLR along with pCMV-Flag-NS5B. The transfected cells were fixed and processed by confocal microscopy using NS5A MAb and rabbit anti-Flag (left panel). The boxed area in the NS5A/Flag-NS5B panel was enlarged (Zoom) to show the colocalization of NS5A with Flag-NS5B (white arrowheads). The degree of colocalization of NS5A with Flag-NS5B was quantified (right panel). (B) Cell lysates from the paired control and hCKα stable knockdown Huh7 cells cotransfected with pTM-NS3-NS5B and pWPI-T7-BLR were subjected to co-IP analysis with anti-NS5A MAb, and the precipitated proteins were analyzed by Western blotting for the indicated proteins. (C to E) The paired stable knockdown cells were cotransfected with pCMV plasmids encoding NS5A-Myc and Flag-tagged NS5B (C), NS3 (D), or NS4B (E). The cell lysates were subjected to co-IP with anti-Myc to determine the binding of NS5A to each of the NS proteins. (F) The paired knockdown Huh7 cells were transfected with plasmids encoding pCMV-Flag-NS4B, pWPI-T7-BLR, and pTM-NS5B. Cell lysates were precipitated with anti-Flag to determine the binding of NS4B to NS5B. ****, *P* < 0.0001.

To support the function of hCKα in the association of NS5A with the viral RC, we first determined the effect of hCKα knockdown on NS5A colocalization with overexpressed Flag-tagged NS5B by confocal microscopy (Fig. 11A, left panel). Depletion of hCKα mitigated the colocalization of NS5A with Flag-tagged NS5B (Fig. 11A, right panel), suggesting the requirement of hCKα for the association of NS5A with NS5B in the viral RC.

To gain insight into the mechanism of hCKα in viral RC formation, we assessed the binding of NS5A with NS5B or NS3 in control and hCKα stable knockdown Huh7 cells expressing NS3-NS5B by co-IP with NS5A MAb. Silencing of hCKα strikingly abolished the interaction of NS5A with NS5B (Fig. 11B). However, hCKα depletion only partially inhibited the binding of NS5A with NS3 as the level of NS3 cocaptured with NS5A was reduced to 43% of that detected in control knockdown cells (Fig. 11B). To dissect the effects of hCKα on NS5A-NS5B and NS5A-NS3 interactions in the absence of other NS proteins, we performed co-IP assays on control and hCKα shRNA stable cells overexpressing NS5A-Myc and Flag-NS5B and overexpressing NS5A-Myc and Flag-NS3, respectively. hCKα depletion greatly interfered with the binding of NS5A to NS5B (Fig. 11C). Nonetheless, the ratio of NS3 to NS5A cocaptured by NS5A MAb was reduced to 0.5 in hCKα-depleted cells compared with that detected in control knockdown cells (Fig. 11D).

Moreover, we performed co-IP using the paired hCKα stable knockdown cells overexpressing NS5A-Myc and Flag-NS4B to understand the involvement of hCKα in the association of NS5A with NS4B. The result showed that hCKα knockdown did not greatly affect the binding of NS5A to NS4B (Fig. 11E). We also examined the effect of hCKα knockdown on the binding of NS5B to NS4B in the paired Huh7 cells coexpressing Flag-NS4B and NS5B driven by the T7 promoter. The result showed that hCKα knockdown did not obviously affect the NS5B-NS4B interaction (Fig. 11F). Taken together, these results indicate that hCKα is indispensable for the stable interaction of NS5A with NS5B and can enhance the binding of NS5A with NS3. However, the NS4B-NS5A and NS4B-NS5B interactions occur independent of hCKα.

### hCKα activity is not essential for the interaction of NS5A with hCKα and NS5B.

**FIG 12:**
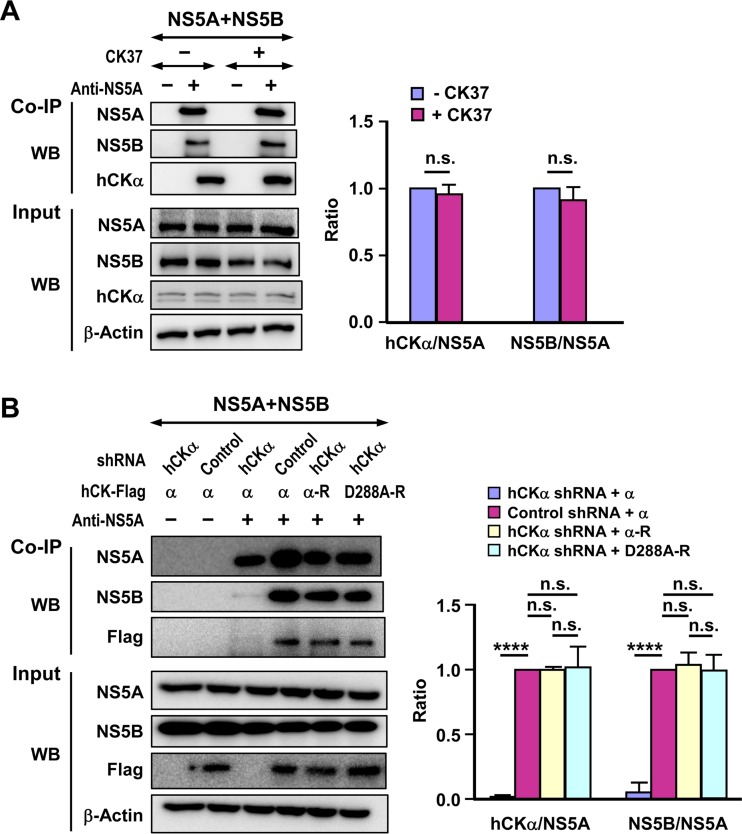
No apparent effect of hCKα activity on NS5A binding to hCKα or NS5B. (A) T7 Pol/Huh7 cells were cotransfected with pTM-NS5A and pTM-NS5B, and cells were treated with DMSO as a negative control or with CK37. Cell lysates were precipitated with isotype IgG or NS5A MAb as indicated, and the coprecipitated proteins were subjected to Western blot analysis for detection of the indicated proteins (left panel). The ratios of hCKα to NS5A and of NS5B to NS5A coprecipitated by NS5A MAb in the CK37-treated cells relative to the ratio detected in vehicle-treated cells, which was arbitrarily set as 1, were quantified. (B) Control and hCKα stable knockdown Huh7 cells constitutively expressing T7 RNA polymerase were transfected with wild-type hCKα (α) or hCKα-R (α-R), or with D288A mutant hCKα-R (D288A-R), followed by cotransfection with pTM-NS5A and pTM-NS5B as indicated. Cell lysates were incubated with isotype IgG or NS5A MAb to determine the binding of NS5A to hCKα or NS5B, respectively. The ratios of hCKα and NS5B to NS5A coprecipitated by NS5A MAb in the experimental setting relative to the ratio detected in control shRNA cells transfected with wild-type hCKα, which was arbitrarily set as 1, were quantified (right panel). ****, *P* < 0.0001; ns, nonsignificant.

Next, we studied whether hCKα activity was crucial for NS5A binding to hCKα and NS5B. T7-Pol/Huh7 cells were cotransfected with pTM-NS5A and pTM-NS5B and then treated with DMSO or CK37. Cell lysates were precipitated with anti-NS5A MAb. CK37 treatment neither affected the NS5A-hCKα interaction nor altered NS5A binding to NS5B (Fig. 12A).

To confirm this finding, the wild-type hCKα or wild-type or D288A mutant hCKα-R plasmid was transfected into T7 RNA polymerase-expressing control or hCKα stable knockdown Huh7 cells, followed by cotransfection with pTM-NS5A and pTM-NS5B. The binding of NS5A to NS5B was greatly impaired in hCKα shRNA cells transfected with wild-type hCKα compared to that detected in control shRNA cells transfected with wild-type hCKα (Fig. 12B), confirming the importance of hCKα in the NS5A-NS5B interaction, as shown in Fig. 11A and B. In addition, the binding of NS5A to hCKα or NS5B was not affected regardless of wild-type or D288A mutant hCKα-R coexpression (Fig. 12B). Together, these findings show that hCKα activity is not involved in hCKα-NS5A binding or in the NS5A-NS5B interaction.

## DISCUSSION

How hCKα impacts HCV replication has not yet been clarified (38, 39). In this study, we showed that hCKα acts as a pivotal proviral regulator to promote viral RNA replication through its enzymatic activity. The concentrations of phosphocholine, the product of hCKα activity, in human liver and milk are 1.4 mM (72) and ∼0.72 mM (73), respectively. However, the addition of up to 2 mM phosphocholine to CK37-treated, HCV-infected cell cultures did not restore HCV replication (data not shown), supporting the hypothesis that hCKα modulates viral RNA replication independent of its production of phosphocholine. In fact, we found that HCV infection and NS3-NS5B expression increased hCKα accumulation on the ER. Although only a small fraction of hCKα colocalized with the viral RC on the ER in both HCV infection and pTM expression systems, this low level of hCKα was sufficient to promote viral RNA replication, as shown by the wild-type hCKα rescue analyses. The incomplete restoration of viral replication mediated by wild-type hCKα-R in the hCKα stable knockdown cells could be due to the low efficiency of DNA transfection into human hepatoma Huh7 cells.

In contrast to our observations regarding the wild-type hCKα, the D288A mutant was poorly localized on the ER, was ineffective at mediating NS5A transport to the ER, and failed to mediate viral RNA replication. Although a basal level of NS5A was localized on the ER when NS5A was expressed in the absence of hCKα, this low level of NS5A had no biological significance for viral RNA replication. These results indicate a critical link between hCKα activity and ER localization of hCKα and NS5A, a critical step in viral RNA replication. In addition, expression of NS3-NS5B increased hCKα localization on the ER, and expression of the wild-type, not the D288A mutant, hCKα enhanced ER localization of NS5A and the hCKα-associated NS5A complex. Moreover, we showed that hCKα was assembled into the viral RC, which might proceed through the interaction of hCKα with D1 of NS5A. These findings suggest that hCKα, via its activity, acts coordinately with NS5A to cooperatively target themselves to the ER where the HCV RC assembles and viral RNA replication occurs. The paradoxical results regarding the hCKα-NS5A interaction obtained by confocal microscopy and co-IP could be due to the fact that confocal microscopy determines colocalization at the subcellular level, whereas co-IP assesses the interaction by concentrating and enriching the two interacting molecules from a large pool of molecules, which makes the co-IP result seem more obvious than the confocal data.

Inhibition of PC synthesis by CK inhibitors in cancer cells often leads to growth arrest and apoptosis (74). Remarkably, siRNA-mediated knockdown or inhibition of the activity of hCKα or CCTα, the rate-limiting enzyme in the CDP-choline pathway, by specific inhibitors did not affect the PC level in mock-infected and HCV-infected cells. These findings indicate that the reduction in viral replication in the HCV-infected cells caused by hCKα knockdown or CK37 treatment is not due to a reduction in the PC level but, rather, to a specific role of hCKα and/or its activity in viral RNA replication. Therefore, our findings indicate that the CDP-choline pathway is not essential for HCV replication.

It is now believed that nonhepatic cells almost exclusively use the CDP-choline pathway for the synthesis of predominantly saturated and nonsaturated PC species, while hepatic cells are unique in their ability to synthesize polyunsaturated PC components through sequential methylation of PE catalyzed by PEMT (Fig. 1A) (75). It was shown previously that the expression of hepatic PEMT and CCTβ2, an isoform of CCTα in mice, was induced in liver-specific CCTα knockout mice (76). In our study, we showed that HCV infection decreased PEMT expression although the cause and significance of reduced PEMT expression in HCV infection are currently unclear. This finding may account for the HCV-induced reduction in the PC level compared to that of mock infection. However, interference with the CDP-choline pathway by transient silencing of hCKα or CCTα in uninfected or HCV-infected cells or by stable hCKα knockdown upregulated PEMT expression, thereby maintaining the homeostasis of the PC pool in cells undergoing impairment of the CDP-choline pathway. As inoculation of cells with UV-irradiated HCV did not affect CCTα or PEMT expression, it is apparent that exposure to UV-irradiated HCV particles may trigger a still unidentified cellular pathway that leads to a reduction in the PC level irrespective of the inhibition of PEMT expression. In support of the notion that UV-irradiated HCV can still bind viral entry coreceptors and trigger a cellular signal, Liu et al. have previously shown that UV-irradiated HCV, as well as untreated HCV, HCVpp, and soluble E2, can induce transient activation of the phosphatidylinositol 3-kinase (PI3K)-AKT pathway and that this activation is mediated by the interaction of the viral E2 with entry coreceptors CD81 and Claudin-1 (77).

**FIG 13:**
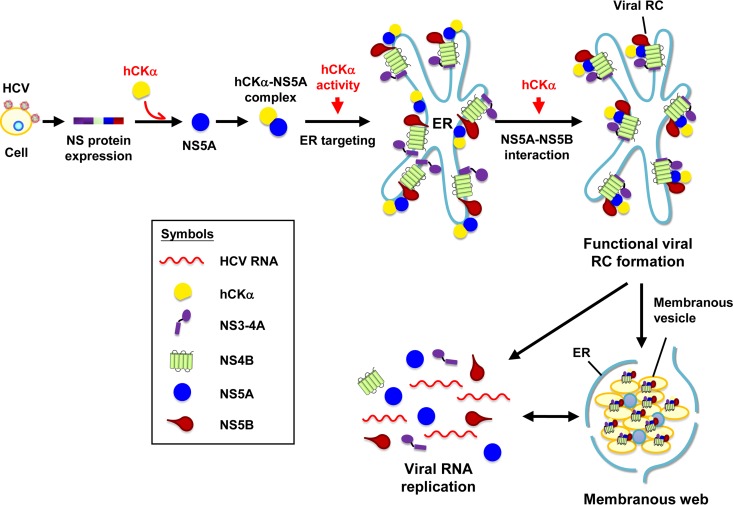
A model illustrating the function of hCKα in HCV RNA replication. HCV usurps hCKα to promote viral RNA replication by facilitating functional viral RC formation on the ER. hCKα is first recruited by HCV, likely via its interaction with NS5A D1; hCKα activity then mediates the targeting of the hCKα-NS5A complex to the ER, whereas expression of NS3-NS5B also enhances the accumulation of the hCKα-NS5A complex on the ER. On the ER membrane, the hCKα protein, *per se*, triggers NS5A and NS5B interactions, resulting in functional viral RC assembly and MW formation. These events subsequently promote viral RNA replication, which subsequently leads to extended MW formation. The steps that involve hCKα activity or protein (not activity) are indicated by red arrows.

As a functional and physical NS5A-interacting counterpart, whether hCKα targets and changes the phosphorylation state of NS5A and therefore modulates viral replication or whether hCKα phosphorylates a still unidentified cellular factor critical for viral replication is currently unknown. Nonetheless, we showed here that hCKα activity participates in MW formation. NS5A localizes in perinuclear membranes such as the ER, Golgi complex, and LDs (9, 78). Alteration of NS5A subcellular localization by an NS5A inhibitor, BMS-790052, prevents the assembly of NS5A into a functional RC and subsequently inhibits viral replication (79, 80), suggesting that the correct localization of NS5A is important for RC assembly. In the present study, we showed that hCKα knockdown inhibited the localization of NS5A, but not NS3 or NS5B, on the ER and mitigated the colocalization of NS5A with NS5B. HCV exhibits a complex network of physical interactions among its encoded NS proteins (81–85). Our co-IP analyses showed that depletion of hCKα significantly interfered with the binding of NS5A to NS5B and only slightly affected NS5A-NS3 binding but did not obviously affect other NS protein-protein interactions. The structure of NS5A may be more accessible by NS5B after complexing with hCKα, and as a result, hCKα triggers the binding of NS5A to NS5B. Nevertheless, hCKα activity was not involved in the binding of NS5A to hCKα or NS5B. Taken together, these results demonstrate the importance of the recruitment of hCKα by NS5A in hCKα activity-mediated targeting of the hCKα-NS5A complex to ER membranes where the hCKα protein, *per se*, acts as regulator to promote the interaction between NS5A and NS5B, leading to functional viral RC assembly and MW formation crucial for viral RNA replication (Fig. 13). However, how hCKα activity mediates the correct targeting of NS5A to the ER warrants further investigation.

NS5A ectopically expressed alone or expressed in the context of NS3-NS5B polyprotein was shown to localize to LDs in Huh7 cells (78). Therefore, in the context of inactive hCKα, we propose that NS5A is mistranslocated to a membrane compartment distinct from, but still juxtapositioned or in close proximity to, ER-derived membranes, such as LDs or other unidentified membranes, and that on the non-ER membrane, the hCKα protein, *per se*, still mediates and stabilizes the binding of NS5A to NS5B. Without the participation of hCKα, NS5A is able to bind NS3, albeit to a lesser extent, but does not efficiently interact with NS5B; NS4B remains able to interact with NS5A and NS5B. In any case, these altered viral RCs are dysfunctional and are incapable of mediating MW formation and viral RNA replication.

Apart from serving as a prognostic marker for cancer progression, hCKα can be used as a molecular target for cancer therapeutics. For instance, RSM-932A, which is being tested as the first in-human drug that targets hCKα, has potent antiproliferative activity *in vitro* and anticancer activity *in vivo* against many different tumor cell lines without toxicity at its effective dose (86). Because of the advancement and use of hCKα-targeted anticancer therapeutic compounds, our study may have important clinical implications for the development of anti-HCV drug therapeutics that target this cellular kinase.
